# Supplementary material for: Cytolinker Gas2L1 regulates axon morphology through microtubule‐modulated actin stabilization
Source: EMBO Rep. 2019 Sep 5;20(11):e47732. doi: 10.15252/embr.201947732 (PMC6831992; doi:10.15252/embr.201947732)
Supplement: Supplementary file 1 — Expanded View Figures PDF [file EMBR-20-e47732-s001.pdf]

Expanded View Figures

Figure EV1. Experimental details of neuronal phenotype validation.

A, B Representative melt curves of various samples amplified with Gas2L1 primers (A) and amplification curves of GAPDH or Gas2L1 cDNA amplified in control and shRNA samples (B) from qPCR experiments shown in Fig 1A. cDNA samples of control (empty shRNA vector, *in duplo*) and Gas2L1-shRNA-expressing neurons (G2L1 shRNA, *in duplo*) show one peak in the melt curve (A), demonstrating specific amplification of Gas2L1 cDNA; negative water control (MQ instead of cDNA template) shows no amplification with Gas2L1-specific primers; negative RNA control (RNA instead of cDNA as template) reveals minor unspecific product that is not amplified and therefore not interfering in the presence of cDNA.

C Fine details of axon morphology of a DIV3 neuron overexpressing HA-Gas2L1 as described in Fig 1B. Boxed regions (1 and 2) are enlarged below. Red asterisk indicates the soma.

D Example of axon tracings for morphology analysis, using the DIV3 neuron expressing scrambled shRNA as shown in Fig 1B as a template. Boxed region is enlarged to the right. Blue tracing denotes the primary axon; the longest possible uninterrupted tracing from the soma to the tip of an axon branch. Red tracings denote non-primary branches and black circle marks the position of the soma.

E–G Rescue experiments showing the total axon length (E), primary axon length (F) and average axon branch length (G) in neurons co-expressing scrambled (Scr) or Gas2L1 shRNA with GFP, or Gas2L1 shRNA with GFP-Gas2L1, and HA-β-galactosidase from DIV0 to DIV3. Data belong to the experiment shown in Fig 1H and I. n = 34–43 neurons per condition (36 for Scr shRNA, 34 for G2L1 shRNA, 43 for G2L1 shRNA + GFP-G2L1) from two independent experiments.

H, I DIV3 neurons overexpressing GFP-G2L1 stained for cortactin (H) or p34-Arc (I), as well as merged images showing co-localization between GFP-Gas2L1 and cortactin (H) or p34-Arc (I) (left panels). Boxes indicate zoomed regions (right panels).

Data information: Scale bars: 25 μm in (C), 30 μm in (D, H, I). Data are displayed as means ± SEM. Mann–Whitney test, ns: not significant, $***P < 0.001$.

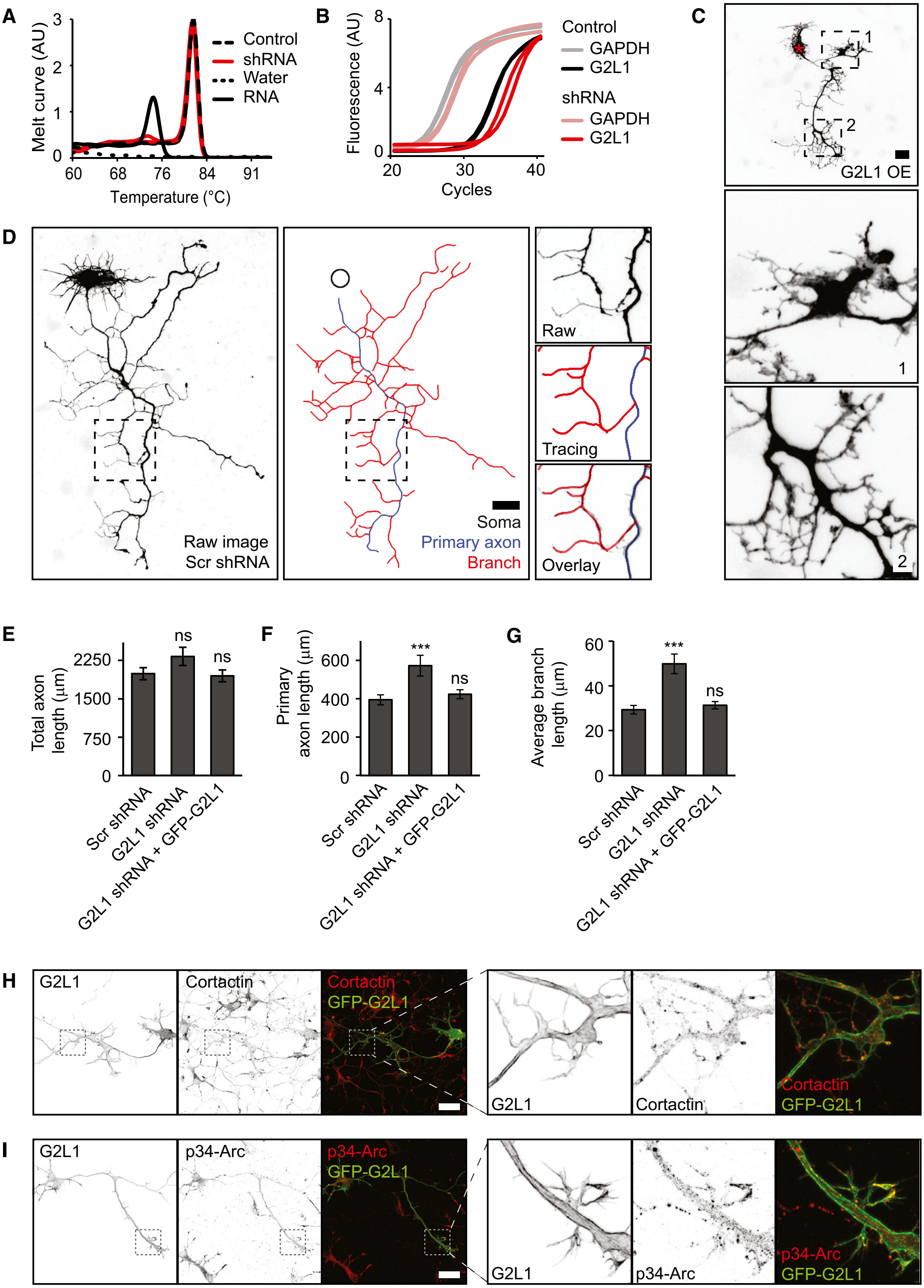

Figure EV1.

 © 2019 The Authors

Figure EV2. ***In vitro*** **reconstitution assays showing the influence of EB3 on the interaction of Gas2L1 with MTs and the relation between Gas2L1 and actin.**

A Coomassie-stained SDS–PAGE gels showing the purity of the purified Gas2L1 (G2L1) fusions used for *in vitro* reconstitutions. For GFP-CH, the additional band at 40 kDa was identified as co-purified actin by Western blotting.

B Intensity ratios of GFP signal over actin signal from Fig 2C (orange) and 2D (green). "Empty" (dark red) is from the channel with only F-actin and no GFP-protein present. This analysis reveals that there is no GFP-Gas2L1 (orange) accumulated on F-actin, since it has nearly the same intensity ratio as the empty channel (dark red). We do observe GFP-CH (green) binding to F-actin. TIRF images were captured on the same day using identical microscope settings.

C, D Kymographs of Gas2L1 (C) and Tail (D) from an *in vitro* reconstitution assay with MTs and EB3. Only the Tail fragment behaves as a plus-end tracking protein.

E–G TIRF images (E) and kymographs (F, G) showing specific Gas2L1 localization to MT-actin overlaps and absence of plus-end tracking in an *in vitro* reconstitution assay with Gas2L1, F-actin (1 μM), MTs and EB3. These data indicate that EB3 does not influence the localization of Gas2L1 in this system, even when EB3 tracks growing MT plus ends (G).

H Control *in vitro* reconstitution experiment showing no alignment of F-actin (1 μM) to MTs in the absence of Gas2L1.

I *In vitro* reconstitution experiments with F-actin (1 μM) without Gas2L1 (top) or with Gas2L1 added (bottom) observed at different time intervals after mixing. Gas2L1 enhances actin bundling and localizes to these bundles. No actin bundles appear at 10 min after flushing in the F-actin mix in the absence of Gas2L1 (top, middle panel), while a few Gas2L1-decorated bundles can be observed right after flushing in the mix in the presence of Gas2L1 (left, bottom panels). Over time, the number of bundles increases as a result of the densification of actin filaments due to the presence of methyl cellulose.

Data information: Scale bars: 10 μm except for (C) and (F). For (C) and (F), horizontal scale bars 5 μm. All vertical (time) scale bars: 3 min, except for (G). For (G), vertical scale bar 5 min.

© 2019 The Authors

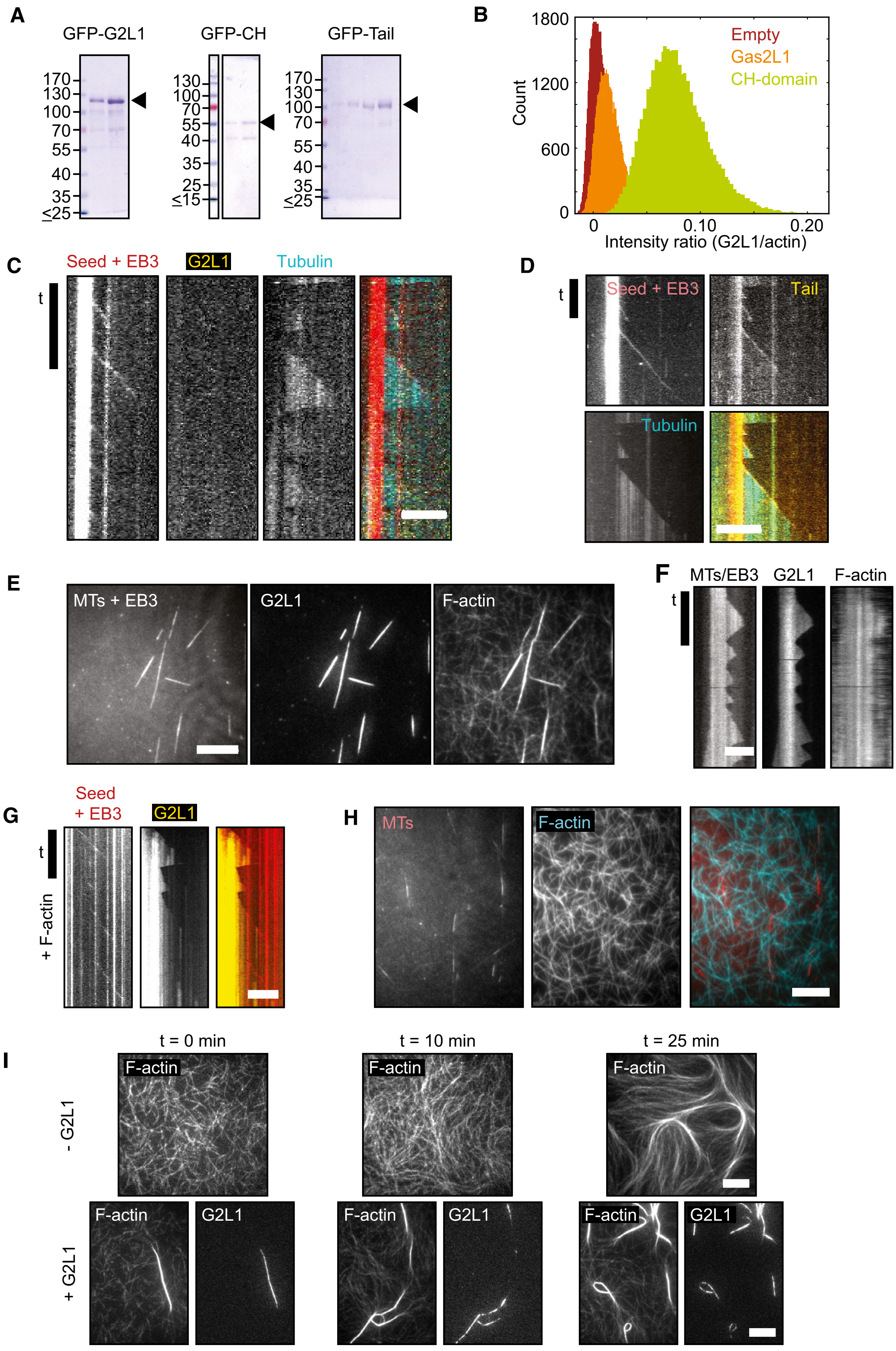

Figure EV2.

 © 2019 The Authors

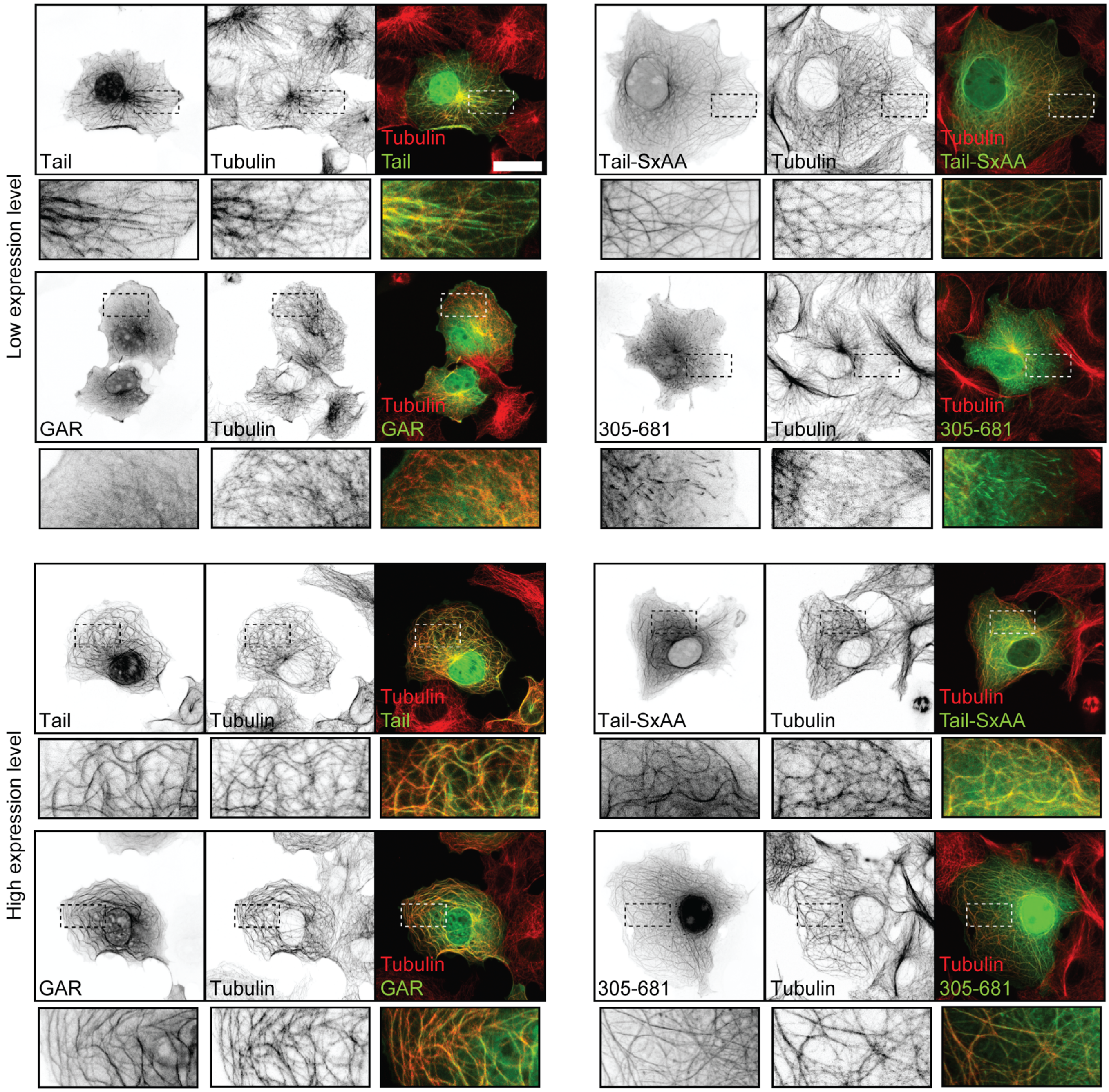

Figure EV3. Localization of Gas2L1 tail domain mutants.

Localization of different Gas2L1 (G2L1) tail domain fragments overexpressed in COS7 cells at low (top panels) and high expression levels (lower panels) and stained for MTs (α-tubulin), as well as merged images showing co-localization of the Gas2L1 fragments with MTs. Bottom panels show enlargements of boxed regions.

Data information: Scale bars: 30 μm.

© 2019 The Authors

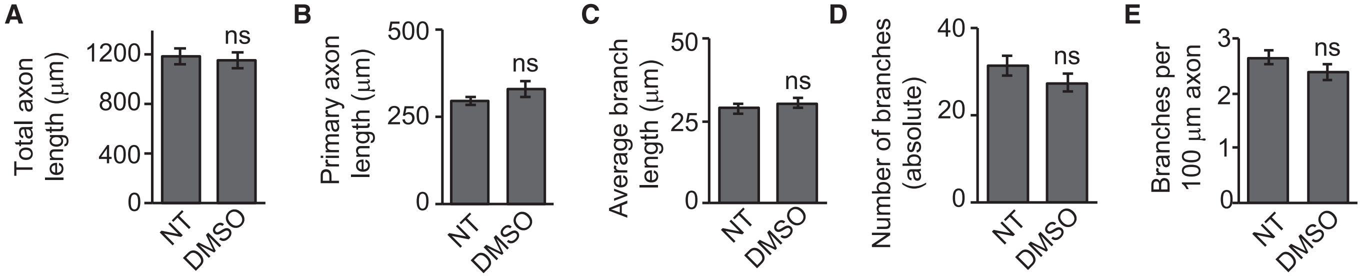

Figure EV4. DMSO does not affect axon morphology.

A–E Effect of DMSO (equivalent to DMSO concentration in 100 nM Latrunculin B condition shown in Fig 5A–E) on axon morphology of DIV3 neurons, transfected at DIV1 and treated with DMSO (1:100) for 48 h. NT = non-treated; *n* = 20–25 neurons per condition (25 for NT, 20 for DMSO) from two independent experiments.

Data information: Data are displayed as means ± SEM. Mann–Whitney test, ns: not significant.

© 2019 The Authors

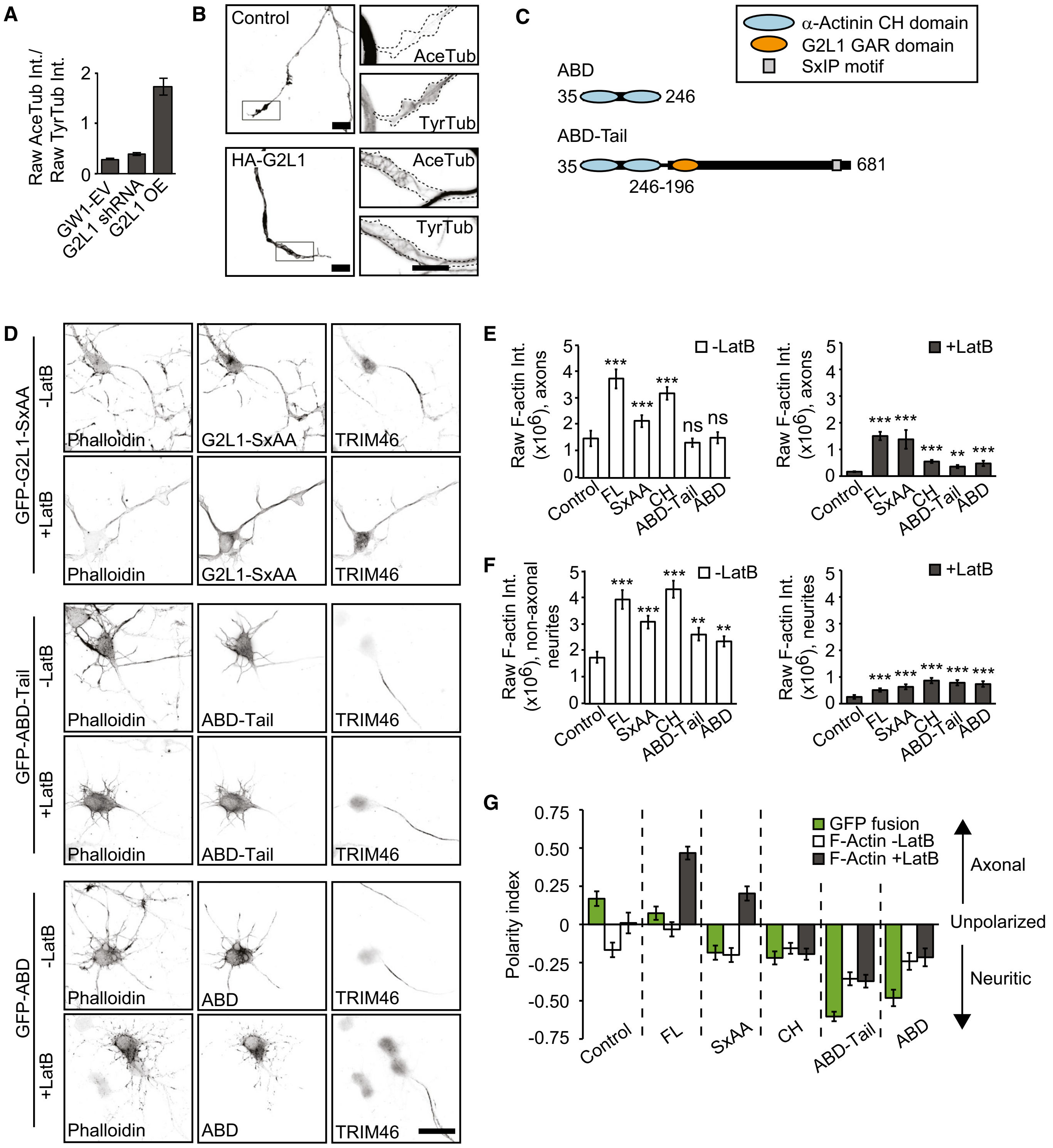

A
Raw AceTub Int./
Raw TyrTub Int.
GW1-EV
G2L1 shRNA
G2L1 OE
B
Control
AceTub
TyrTub
HA-G2L1
AceTub
TyrTub
C
α-Actinin CH domain
G2L1 GAR domain
SxIP motif
ABD
35
246
ABD-Tail
35
246-196
681
D
GFP-G2L1-SxAA
-LatB
+LatB
Phalloidin
G2L1-SxAA
TRIM46
GFP-ABD-Tail
ABD-Tail
GFP-ABD
ABD
E
Raw F-actin Int. (x10^6), axons
-LatB
+LatB
Control
FL
SxAA
CH
ABD-Tail
ABD
ns
F
Raw F-actin Int. (x10^6), non-axonal neurites
Raw F-actin Int. (x10^6), neurites
G
Polarity index
GFP fusion
F-Actin -LatB
F-Actin +LatB
Axonal
Unpolarized
Neuritic

Figure EV5.

Figure EV5. The MT-binding tail fragment does not influence localization of a different actin-binding domain.

A Quantification of the ratio of raw acetylated tubulin (AceTub) intensity to raw tyrosinated tubulin (TyrTub) intensity in growth cones of DIV4 neurons transfected with GW1-EV, G2L1 shRNA or HA-G2L1 (G2L1 OE) and HA-β-galactosidase for 1 day. *n* = 56–85 growth cones from 37 to 43 neurons per condition (68 growth cones from 43 neurons for GW1-EV, 56 growth cones from 37 neurons for G2L1 shRNA, 85 growth cones from 41 neurons for G2L1 OE) from three independent experiments.

B Silhouettes (from β-galactosidase fill, left panels) of growth cones of DIV4 neurons, transfected with GW1-EV (control, top panels) or HA-Gas2L1 (bottom panels) and HA-β-galactosidase. Boxes indicate zoomed regions (right panels), showing staining for acetylated tubulin (AceTub) and tyrosinated tubulin (TyrTub). Dotted lines show the outline of the growth cone.

C Schematic overview of α-actinin mutants used in (D–G).

D Additional conditions included in the experiment shown in Fig 6F: DIV3 neurons expressing GFP-ABD or GFP-ABD-Tail and treated with 10 μM Latrunculin B for 30 min or non-treated, stained for F-actin (phalloidin) and the axon initial segment (TRIM46).

E, F Additional conditions included in the experiment shown partially in Fig 6G and H: raw phalloidin staining intensities in axons (E) and non-axonal neurites (pre-dendrites) (F) of DIV3 neurons expressing the indicated GFP fusions and treated with 10 μM Latrunculin B for 30 min (grey bars) or non-treated (white bars). *n* = 27–40 neurons per condition (40 for GFP, GFP-G2L1, GFP-CH, GFP-ABD-Tail, 35 for GFP-SxAA, 27 for GFP-ABD) from two independent experiments.

G Additional conditions included in the experiment shown partially in Fig 6I: polarity index representing localization of the indicated GFP fusions in non-treated neurons (green bars), and localization of F-actin as labelled by phalloidin in DIV3 neurons treated with 10 μM Latrunculin B for 30 min (grey bars) or non-treated (white bars). *n* = 27–40 neurons per condition (40 for GFP, GFP-G2L1, GFP-CH, GFP-ABD-Tail, 35 for GFP-SxAA, 27 for GFP-ABD) from two independent experiments. Positive values indicate axonal enrichment, negative values indicate neuritic enrichment, and a value of 0 signifies no particular enrichment.

Data information: Scale bars: 10 μm in (B), 30 μm in (D). Data are displayed as means ± SEM. Mann–Whitney test, ns: not significant, $^{**}P < 0.01$, $^{***}P < 0.001$.

© 2019 The Authors
